# The Glycemic Index and Human Health with an Emphasis on Potatoes

**DOI:** 10.3390/foods11152302

**Published:** 2022-08-02

**Authors:** Venkata Sai Sagili, Priyadarshini Chakrabarti, Sastry Jayanty, Hemant Kardile, Vidyasagar Sathuvalli

**Affiliations:** 1 Department of Integrative Biology, 3029 Cordley Hall, Oregon State University, Corvallis, OR 97331, USA; sagiliv@oregonstate.edu; 2 Department of Biochemistry, Molecular Biology, Entomology and Plant Pathology, Mississippi State University, 100 Old Highway 12, Mississippi, MS 39762, USA; pb1090@msstate.edu; 3 San Luis Valley Research Center, Department of Horticulture and Landscape Architecture, Colorado State University, 0249 East Road 9 North Center, Fort Collins, CO 81125, USA; sastry.jayanty@colostate.edu; 4 Department of Crop and Soil Science, 109 Crop Science Building, Oregon State University, Corvallis, OR 97331, USA; kardileh@oregonstate.edu; 5 Hermiston Agricultural Research, and Extension Center, Oregon State University, 2121 South 1st Street, Hermiston, OR 97838, USA

**Keywords:** glycemic load, obesity, diabetics, carbohydrate-rich foods, resistant starch

## Abstract

Diabetes and obesity are associated with the excessive intake of high-glycemic index (GI) carbohydrates, increased glycemic load (GL) foods, and inactive lifestyles. Carbohydrate-rich diets affect blood glucose levels. GI is an indicator of the impact of a specific food on blood glucose, while GL represents the quantity and quality of carbohydrates in the overall diet and their interactions. There are in vitro and in vivo methods for estimating GI and GL. These values are useful human health markers for conditions such as diabetes, obesity, and pregnancy. Potato is a major starchy vegetable, which is consumed widely and is the fourth most important crop globally. However, the GI of diets rich in starchy vegetables such as potatoes has not been studied in detail. The GI values in potatoes are affected by external and internal factors, such as methods of cooking, methods of processing, resistant starches, cultivation methods, mixed meals and food additions, and hormone levels. This review summarizes how these factors affect the GI and GL associated with diets containing potatoes. Understanding the impacts of these factors will contribute to the development of new and improved potato varieties with low GI values. The consumption of low-GI foods will help to combat obesity. The development of low-GI potatoes may contribute to the development of meal plans for individuals living with diabetes and obesity.

## 1. Introduction

The glycemic index (GI) is a measure of the blood glucose response to foods [1], while the glycemic load (GL) is the product of the GI and the total available carbohydrate content in a given food [2]. Foods have been classified according to their GI values. Carbohydrate-rich foods that are easily digested, absorbed, and metabolized have a high GI (GI ≥ 70 on the glucose scale), while low-GI foods (GI ≤ 55 on the glucose scale) contain slowly digestible carbohydrates that elicit a reduced postprandial blood glucose response [1]. Intermediate-GI foods have a GI between 56 and 69 [2]. A food with a higher GI value raises the blood glucose level faster than a food with a lower GI value [1]. Starch is the major component of carbohydrate-rich foods obtained from major cereal crops, such as rice, wheat, maize, and barley. Additionally, there are vegetables (potatoes, casava, and yams) which are rich in starch; these are often called starchy vegetables [3]. Among these starchy vegetables, potatoes, a moderate- to high-GI food [4], are a dietary staple for 1.3 billion people in the world. Potato is the world’s fourth most important crop after rice, wheat, and maize, and the major food crop among non-grains. Because they are easy to cook and highly palatable, potatoes are a predominant component of meals and snacks in the food industry [5].

There are a number of food options and menu choices globally which include potato as a major vegetable, and the GL of these foods can vary depending on many factors. In a human health context, GI/GL values are useful because they can act as satiety markers. Blood glucose levels are an important metabolic marker for diabetic patients [1]. As a high-GI food, potato is viewed as a less healthy dietary option; however, there are many aspects to consider about the value of potato in the human diet. Post-harvest processing can have a major effect on the GI/GL values of potatoes. For instance, the various cooking processes, such as baking, microwaving, boiling, and frying, can affect the GI values of potato-containing diets [6]. Further food additives can affect the GL. 

This review summarizes the concepts of GI and GL and how their values are estimated and discusses the factors affecting the GI/GL of potato. It also covers the implications of GI/GL on human health and ways to develop improved potato varieties with low GI values. 

## 2. GI Estimation

There are in vivo and in vitro methods of calculating the GI values of foods, as reviewed in detail by Lal et al., 2021 [7]. To study the GI values of foods using human subjects, one must measure the subjects’ blood glucose levels before consuming a food, and then again after consuming it. Foods that raise the blood sugar levels more quickly are assigned a higher GI value. The problem with many in vivo calculations of GI is that the biological factors affecting GI values vary among different people. In one study testing the reliability of GI calculations for white bread, insulin index and glaciated hemoglobin values explained up to 15% of the difference in GI values between subjects [8]. Higden et al. presented the most common in vivo method of calculating GI [1]. In vitro methods of calculating GI values attempt to replicate the human digestion process at the bench scale. This process comes with its own benefits and drawbacks. On the one hand, the lab process can be standardized more simply than in vivo methods, which always involve variability among the test subjects. However, any attempt to replicate the human digestion process will differ from an actual human body. Measuring the GI of foods using in vivo and in vitro methods allows us to compare and contrast the validity of results. In recent years, many in vitro GI protocols have been developed [9,10].

Despite the variability in calculated GI values, it is possible to establish a general trend determining which foods have a low or a high GI, and their implications for human health can be explored. GI values of foods are calculated using the following formula [1]:

GI = (IAUC test food ÷ IAUC glucose) × 100(1)

where IAUC = the incremental area under the curve.

The IAUC is calculated according to the trapezoidal rule for calculating area. The trapezoidal rule is a method of calculating area under the curve using calculus by approximating the area under the curve as trapezoids. The curve itself is determined by measuring the blood glucose over time.

The food IAUC is an increment in the test food area under the curve. The glucose IAUC is an increment in the reference food area under the curve. 

## 3. GL Estimation

The glycemic load (GL) is a useful supplementary measure to the GI. The GL measures the quality and the quantity of a carbohydrate in a meal [1]. High GL values are typically above 20, intermediate values are between 11–19, and low values are under 10 [1]. The GL of a food is calculated based on the formula as follows [1]:

GL Food = ((GI Food × amount (g) of available carbohydrate food per serving) ÷ 100)(2)

The calculation of the GL includes the quantity of a carbohydrate ingested. For example, watermelon has a mean GI of 76, which is the same value as a doughnut, although one serving of watermelon has 11 g of carbohydrates and a doughnut has 23 g [1]. However, one serving of watermelon has a GL of 8 and that of a doughnut is 17 [1]. One recommendation for the adoption of GI and GL values in daily life would be to publish these values on the nutritional facts labels of foods, along with the calories and ingredients. This would allow consumers to make informed decisions about the GI and GL values of the foods they are eating.

## 4. Glycemic Index in Potatoes

Starchy vegetables are a staple food that can be used to mediate GI intake [2]. Even though starchy vegetables have varying GI values, in general they have higher GI values than non-starchy vegetables. This could be due to the relationship between the starch availability and the enzyme amylase, which makes food more easily digestible [11]. Amylase is a digestive enzyme that breaks down carbohydrates [11]. Many starchy vegetables, such as potatoes and yams, are part of staple diets throughout the world. It is important to note that even among the starchy vegetables, there is a wide range of GI values based on the breed of the potato, yam, etc. [12]. The cultivated potato (*Solanum tuberosum* L.) is the most widely consumed vegetable and a critical staple food in many developing countries. Potatoes are not only an important fresh-market crop but also a raw material for French fries, chips, and the starch-processing industries. The potato is grown in over 150 countries, with the employment of ~205 million people across the entire production chain. Because of its worldwide production and utilization as a major food staple, the potato contributes to the United Nation’s Millennium Development Goals to enhance food security and eradicate poverty. As a major crop with the potential to solve food insecurity, it is essential to understand the GI values of potatoes. Furthermore, understanding the GI values of potatoes will be crucial for establishing balanced, healthy diets [5]. Consuming lower-GI potato varieties or potato foods with low GL values could have a markedly positive effect on the health outcomes of Americans and people around the world. In addition, consuming potatoes with low GI values can help to control the blood sugar, regulate diabetic conditions, and lower cholesterol, triglycerides, and overall weight [1]. Low-GI potato diets can lead to fewer metabolic markers associated with inflammation—including proteins such as the C-reactive proteins [1]. The GI values of different potatoes and potato products are presented in Table 1. Some potato varieties, such as the ‘Maris Peer’, are higher in GI value, while other varieties, such as ‘Marfona’ or ‘Nicola’, have low GI values. This suggests that the varieties such as ‘Marfona’ or ‘Nicola’ might be considered as replacements for higher-GI-value potato varieties in the diets of those who want to control their blood sugar. Apart from genotypes, there are additional factors that affect the GI values in potatoes.

## 5. Factors Affecting the GI Values of Potatoes

**Figure 1:**
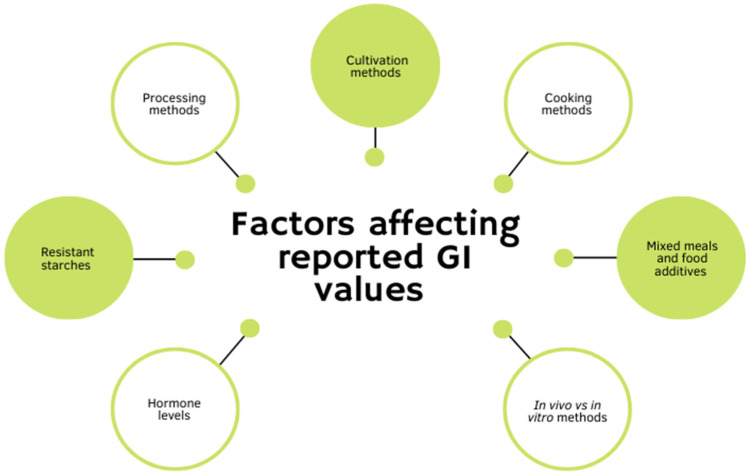
Factors affecting reported GI values.

While much research has been conducted on the importance of the GI of foods, there is a gap in knowledge on the actual GI values of many foods [14]. There is little standardization among the published GI values of various foods, even if they have the same names or descriptions. Additionally, the interactions between the numerous factors that affect GI values are not well understood (Figure 1). Some of these important factors that affect the GI values in potatoes are discussed below.

### 5.1. Cooking Method

Raw potato tubers are not consumed in the human diet. To study the effect of potato GI on human health, potatoes must be either cooked or otherwise processed. Cooking impacts GI/GL of potatoes and other properties. Cooked potato is generally considered as a high GI food because of its high content of rapidly digestible starch [15]. Domestic cooking methods, such as boiling, frying, steaming, microwaving, and roasting, are usually adopted for potato-cooking throughout the world. These cooking methods modify the chemical, physical, and enzyme changes to the tuber starch content, eventually affecting the GI. Approximately 90% of the potato’s dry weight mass is starch, which is divided into digestible starch (DS) and resistant starch (RS) based on digestibility [16]. It has been demonstrated that the consumption of RS is negatively correlated with high postprandial blood levels [17]. Heating during the domestic cooking of potatoes leads to gelatinization and the formation of RS. Cooking processes also result in physical changes which change the microstructure of the starch. Mashed and boiled potatoes have higher GIs than fried, microwaved, or baked potatoes, primarily because of the degree of gelatinization and physical changes to the microstructure caused by these cooking methods [6]. Frying is the most frequently used cooking method in quick-serve restaurants. Frying leads to complete starch gelatinization in the internal parts of the potato, while on the surface, the high temperature leads to the formation of lipid–amylose (RS). By contrast, boiling results in the complete collapse of potato cell, as well as swelling and gelatinization of the intracellular starch, making the starch easier to digest. It has been reported that boiling, microwaving, baking, and deep-fat frying alter the RS content of potatoes by 2.9%, 7.3%, 6.2%, and 9.1%, respectively [18]. The temperature and cooking methods (boiling, roasting, and frying) result in varying GI values of potato (Table 1). Baked tubers showed a significant reduction in GI values as their resistant starch (RS) values increased when stored at 4 °C after cooking [14]. Higher RS values tend to elicit a lower GI response [19]. Cooking with various methods can elicit different RS changes in potatoes. According to one study, baking and steaming appear to increase the amount of RS in foods, while pressure cooking decreases the amount of RS [20]. Thus, when picking a method for cooking potatoes, people who want to keep their GI in mind might consider baking as one of the better options.

### 5.2. Method of Processing

Processing methods can also impact food GI values. Highly processed carbohydrates tend to have higher GI values (Table 1). In general, potatoes and corn chips have higher GI values than many of minimally processed starchy vegetable foods [21]. Potatoes processed for French fries tend to have a higher GI compared to potatoes processed for chips. The difference between the GI values of chips and fries is associated with the availability of RS. The mealiness of the French fries is a major contributor to the increased GI in contrast to chips. Additionally, roasting or baking certain foods result in higher GI than boiling [22].

### 5.3. Resistant Starches

Resistant starches are not digested in the small intestine, but rather are fermented in the large intestine, where they play a role similar to dietary fiber and induce a lower GI response [19]. Generally, foods containing more amylose are more resistant to digestion. There are five major types of resistant starches, which elicit varying degrees of GI response. The major types are RS-I to RS-V. RS-II is predominant in uncooked potatoes. Processing and cooking increase the digestibility of RS-II. Environment, biotechnology, and natural mutations can affect the starch structure and its chemical properties that influence resistance to digestion [11]. We found that there is variability among the cultivars for resistant starch, and the baked tubers have low amounts of resistant starch when compared to the raw tubers (Table 2). This suggest that baking potatoes lowers the amount of RS; thus, research on which methods of cooking reduce RS the least, and their effect on RS-II, may be future areas for exploration.

### 5.4. Cultivation Methods

Crop production practices and edaphic factors, such as soil type, soil organic matter content, etc., warrant further research, as they may impact GI values [11]. There are few studies, if any, that consider how factors such as soil health impact GI. Healthy soils are an important factor in food production—the soil in which crops grow is an ecosystem in and of itself, which is full of living macro- and micro-organisms that can affect plant growth and quality [24]. Many studies indicate that soil health can affect crop yields and quality, but little has been written about the connection between soil health and GI. For example, a highly polluted, non-fertile soil has been shown to produce significantly lower yields than healthy soil [24]. In another study, a maize crop irrigated with polluted river water produced a 25% lower yield than maize grown with potable well water [25]. The field production environment can change the thermal properties of crop starch content. Current theories propose that the environmental temperature alters the function of certain starch enzymes inside the crops [11]. Further research in this area would support the development of various crops with increased resistant starch content. The agroclimate of the environment has important effects on the crops grown. For example, the *terroir* effect in grape vines plays an important role in wine typicity [25], indicating that soil factors such as water availability, plant nutrients, temperature, and moisture have marked effects on grape vines [25]. These differences are manifested in the wine, and there are significant differences in taste, as measured by sensory evaluation [25]. Similar soil factors could impact the factors relevant to potatoes, such as amylose content. Growing conditions have an impact on tubers, especially certain tubers such as the ‘Desiree’ or ‘Kuras’ varieties [26]. Another example of a biotic factor or growing condition which affects GI values is the potato apical leaf curl disease, which can affect GI values [27]. This disease is a stressor which affects the synthesis of carbohydrates, proteins, and starch, which in turn affect the GI of the resultant tubers [27]. 

### 5.5. Mixed Meals and Food Additivies

GI values can also vary based on the total meal nutrient composition [28]. While GI values are calculated for individual food items, eating patterns tend to be more complex, with multiple foods consumed during a single meal [28]. These combinations of foods can impact the GI response [28]. One study found that individuals who ate mixed meals containing all three macronutrients—carbohydrates, proteins, and fats—exhibited a decrease in the GI value impact beyond the calculations performed on the individual foods [28]. The implications of this preliminary study suggest that complex interactions among nutrients digested together affect the GI value. Thus, one should consider including multiple macronutrients, such as proteins and fats, in one’s meals rather than focusing on a single macronutrient. Proteins may produce this effect by changing the amount of insulin secreted in response to increased blood sugars, especially in diabetic patients [29]. In some parts of the world, potato is consumed with other vegetables or cereals. These combinations, too, affect the overall GI and GL of a meal. The effects of the combination of potato with eight different types of vegetables were studied. Fresh market and processing cultivars were examined for their predicted GI, GL, RS, and related parameters. The addition of vegetables to potatoes resulted in a significant reduction (*p*  <  0.05) in GI with an increase in the RS content of the combined food material. It was also discovered that eight different vegetables taken in combination with potato resulted in a lowering of the GI and GL by up to 20 and 42%, respectively. Among the vegetables, amaranthus, spinach, and eggplant were the least effective in lowering the glycemic response, and fenugreek leaf, fenugreek seed, cauliflower, okra, and bitter gourd were most effective [30].

Food additives have a direct impact on GI. Food additives such as sugars, butter, cream, etc., when added to starchy foods like potatoes, will have a negative impact on GI. When considering the addition of sugars or other food additives, it is essential to understand their impact on GL. Here, we provide a brief review of honey as a food additive and its impact on GI/GL. 

Honey is a common replacement for cane sugar as a sweetener in beverages and baked goods. It is also recommended as an alternative natural sweetener for diabetic patients [31]. Unlike cane sugar, honey contains over 200 compounds, including vitamins, minerals, and phytochemicals in addition to fructose, glucose, and water, which are the majority of its constituents [32,33,34]. Floral sources determine the sugars in honey; presumably they also determine the GI and GL values [32]. The content of monosaccharide sugars such as glucose and fructose, can also vary. For example, fructose content in honey can range from 21% to 43% and the ratio of fructose:glucose can vary from 0.4 to 1.6 or more [31,35,36,37]. The GI value of fructose is 19, while the GI value of sucrose is 60 and the value of glucose is 100 [38]. Thus, when evaluating the GI and GL values of honeys, a range of values is quite possible [39]. The 2008 International Tables of GI and GL Values indicate that honey has a GI of 61 ± 3 [14]. Some older studies list the ranges from 32–87 or even from 58–87 [39,40]. An Australian study found that bees foraging on yellow box, stringybark, red gum, iron bark, and yapunyah trees produced low GI-honeys, whereas salvation Jane and commercial honey blends had moderate to high GI values [32]. Another study in Jordan showed that honey produced by bees foraging on Christ thorn, citrus and locust produced honey with GI values lower than sucrose [41]. The GI values of manuka honey can range from 54–59 [42]. In Turkey, the GI values of honeys produced by bees foraging on five common crops were found to be as follows: citrus, 44.9, thyme, 52.6, lime, 55.3, chestnut, 55.5, pine, 58.8, and milk-vetch, 69 [43]. Thus, honey can offer an alternative to many sugars or sweeteners, depending on its floral sources. The GI values of different types of honey are presented in Table 3. Thus, the sources of food additives can impact the overall GL of the foods we eat.

### 5.6. Hormone Levels

Not all carbohydrates are equal in their assigned GI values. Low-glycemic index carbohydrates, on average, lead to increased satiety, but the mechanisms behind this effect remain unclear. One proposed mechanism is that low-GI meals affect the gut hormone levels associated with satiety [44]. For example, GLP-1, an appetite-suppressing hormone, is found in higher levels in the bloodstream following the consumption of a low-GI meal [44]. GLP-1 operates synergistically on the central and peripheral receptors associated with appetite; it regulates the appetite in both the gut and the brain [45]. Whole wheat breads, which have lower average GI values than white breads, have been shown to induce greater GLP-1 production than white breads [44]. Recently, there has been a boom in research regarding the gut microbiome; thus, studies on the levels of hormones and bacteria in the stomach could lead to important discoveries regarding GI in the near future.

Potatoes, specifically, have an impact on GLP-1 when eaten in a “mixed meal” such as those mentioned in Section 5.5 [46]. GLP-1 levels were lower when potatoes were consumed with a high-fat meal, and higher when the high-fat meal was eaten without potatoes [46]. This provides evidence that, consumed together, mixed meals and potatoes both have positive effects on satiety and the release of GLP-1, which can be useful when trying to combat obesity through diet. When a person feels fuller, they are less likely to feel the need to eat more food and exceed the proper calorie count.

### 5.7. In Vitro vs. In Vivo Measures

The method of measuring the GI can result in different values. In general, the main issue regarding in vivo vs. in vitro testing is that in vitro testing will never be able to fully replicate the digestive system of the human body, including features such as the peristalsis of the gastrointestinal tract [47]. In vitro methods attempt to replicate the human digestive system through appropriate enzyme blends and protocols including shaking, but, ultimately, in vivo methods will still likely provide the most accurate GI value results at the present time. Furthermore, as explained in Section 5.6, the gut microbiome and associated digestive hormones vary among individuals and affect the in vivo GI values. 

## 6. Potato Consumption Habits of the Next Generation and the Impact of GI on Human Health

The next generation of Americans, or “Generation Z”, have their own developing food values and preferences. They value health, convenience, and sustainability more than previous generations. Half of Generation Z consumers reported that they would pay more for healthier foods, while only 32% of Millennials did [48]. Plant-based diets are becoming more common [49]. Potatoes and other starchy vegetables are poised to play an essential role in Generation Z’s lifestyle because they are plant-based, can be cooked conveniently, and have significant health benefits. Food habits and food preferences affect human health. An understanding of the influence of the GI and GL values of foods would provide helpful guidance in making food choices. Health benefits should, therefore, be a focus in plant-breeding efforts. Low GI can be a marker of more healthy potatoes; thus, the breeding of lower GI potatoes would likely have market appeal among Generation Z members.

GI values can be informative in a clinical setting. The U.S. obesity epidemic is associated with diabetes and heart disease [50]. GI values could become one tool among many to guide healthy food choices and combat obesity. Low-GI foods provide a longer-lasting feeling of satiety and reduce the overall amount of food consumed. Low-GI diets have been used for years as an intervention in patients with diabetes to regulate blood glucose levels. Meta-analysis over decades of study on this subject has shown that low-GI diets can offer a useful intervention for those with diabetes or pre-diabetes [51].

## 7. Developing Low-GI Potatoes

The idea of breeding crop plants such as potatoes for various traits, such as yield and hardiness, has been explored since people started farming, but only recently have humans begun to breed plants for their nutritional values [52]. Genomics and genetic engineering are cutting-edge technologies, which support trait-specific crop improvements.

Modern technology accelerates the breeding and development of potatoes with low GI values. In vitro and in vivo experiments can pinpoint the GI values of different potato varieties. Modern genomic technologies can identify the genetic markers associated with low GI values. This genetic/genomic information can be used to select progenies with the potential for reduced GI values. Various studies have identified genetic markers associated with potato starch [3,53]. Similarly, genomic regions associated with low GI in potatoes can be identified and employed in marker-assisted breeding.

Mutations in plants can lead to changes in GI values. Genetic loci in maize encode starch-branching enzymes, and mutations in these areas have resulted in starch with greater amylose, which is associated with greater starch resistance to digestion and therefore, potentially, reduced GI values [54]. The identification of more such genotypes will help to improve breeding. For example, starch that is modified to include 100% amylose is highly resistant to digestion [10]. In potatoes, variations in the amylose and amylopectin values have been observed among varieties (Table 4). This indicates that natural variation within the potato germplasm is existent and can be leveraged to breed for lower GI potatoes.

The genetic study of the wild relatives of crops as sources of breeding material is an area of interest to plant breeders. Certain low-amylopectin-content potato genotypes that exist in the wild may have been used by indigenous people to combat blood sugar/obesity [55]. In modern-day cultivars, the potato breeds with the lowest amylopectin contents were found in ‘Huckleberry Gold’, ‘Muru’, ‘Multa’, and ‘Green Mountain’ varieties [55]. There are many ways to screen for amylopectin content, including the examination of granule structure, water absorption, and spectrophotometry of the iodine complexes [55]. Once the major biochemical and structural traits that impact the GI of potatoes are identified, it will become possible to screen the germplasms for low-GI traits, and to use such potatoes in the breeding programs. The genetic markers and identification of genomic regions linked to low GI will enhance the breeding of low-GI potato varieties. Biotechnological tools such as genetic engineering and gene-editing can also promote the development of low-GI potatoes.

## 8. Conclusions

Obesity is a global epidemic. Many chronic illnesses and diseases, such as diabetes are associated with obesity [50]. Ensuring that nutritious food is available and affordable to the public is critical for combatting this epidemic. Starchy vegetables such as potatoes are an essential part of the global diet. GI has emerged as an important nutritional factor in starchy vegetables, and the consumption of low-GI foods can act as an important tool in combatting obesity. To date, the focus of starchy vegetable breeding and their production revolves around increasing the yield and crop resilience to biotic and abiotic factors, rather than on health-attributing traits such as GI. There are many factors that directly impact the GI values of potatoes. Given their importance in the global diet, further research into the calculation of GI values and the factors that affect GI is warranted. New advances in technology and genomics will play an essential role in breeding potatoes for lower GI values. An increased focus on breeding for low GI using the genomic methods should be explored.

## Figures and Tables

**Table 1:** GI of potatoes and potato-based food products.

Type of Potato Product and Cooking Method	GI Value	Reference
‘Maris Peer’	94	[13]
‘Maris Piper’	85	[13]
‘Desiree’	77	[13]
‘Estima’	66	[13]
‘Charlotte’	66	[13]
‘Marfona’	56	[13]
‘King Edward’	75	[13]
‘Nicola’	59	[13]
Russet, baked	111	[1]
Potato, white, boiled (average)	82	[1]
California white potato, roasted	72.3 ± 8.2	[12]
Boiled red potato, hot	89.4 ± 7.2	[12]
Boiled red potato, cold	56.2 ± 5.3	[12]
French fries	63.6 ± 5.5	[12]
Chips/crisps	56 ± 3	[14]
Rice, white, boiled ^1^	66	[1]
Rice, brown, boiled ^1^	50	[1]

^1^ Values are shown as per the reference.

**Table 2:** Estimated total starch and resistant starch in Colorado-grown potatoes (raw and baked).

Cultivar	Potato Type	Total Starch (g/100 g)	Resistant Starch ^1^ (g/100 g Total Starch)		Non-Resistant Starch (g/100 g Total Starch)
			RS-Raw	RS-Baked	
Purple Majesty	Specialty	70.46 (±0.2)	13.43 (±1.8)	3.24 (±1.2)	96.86 (±12)
Yukon Gold	Yellows	60.10 (±1.2)	34.64 (±4.0)	2.32 (±1.4)	97.73 (±2.2)
Rio Grande Russet	Russets	59.84 (±5.5)	23.71 (±6.0)	9.70 (±1.3)	91.16 (±1.3)
Rio Colorado	Reds	63.32 (±1.5)	17.98 (±3.2)	3.73 (±3.0)	96.40 (±1.1)
Mountain Rose	Specialty	62.16 (±1.8)	12.15 (±1.4)	6.71 (±2.9)	93.72 (±2.3)
Lenape	Chip	63.90 (±2.0)	14.52 (±1.8)	6.14 (±1.8)	94.22 (±2.1)
CO94035-15RU	Russet	68.49 (±2.1)	20.27 (±4.0)	5.33 (±1.11)	94.94 (±1.1)
CO95051-7W	Chip	71.81 (±1.1)	32.86 (±5.4)	5.72 (±2.3)	94.59 (±2.2)
AC96052-1RU	Russet	66.37 (±1.6)	14.62 (±1.5)	10.38 (±1.3)	90.60 (±11)
CO97226-2R/R	Specialty	60.48 (±2.8)	9.81 (±1.1)	8.77 (±2.8)	91.93 (±0.9)
CO97232-1R/Y	Red	66.00 (±6.0)	23.78 (±3.2)	5.49 (±3.2)	94.80 (±1.1)
AC97521-1R/Y	Red	61.02 (±3.2)	23.76 (±1.2)	7.07 (±1.9)	93.39 (±1.8)

^1^ Measurement methods are available from Jayanty et al., 2007 [23].

**Table 3:** GI values of different types of honey.

Honey Source	GI	Reference
Manuka	54–59	[42]
Citrus	44.9 ± 15	[43]
Thyme	52.6 ± 20.1	[43]
Lime	55.3 ± 18.4	[43]
Chestnut	55.5 ± 20.2	[43]
Pine	58.8 ± 27	[43]
Milk-Vetch	69 ± 27.3	[43]
Yellow Box	31–39	[43]
Stringybark	40–48	[32]
Red Gum	43–49	[32]
Iron Bark	45–51	[32]
Yapunya	47–57	[32]

**Table 4:** Variation of amylose content in potato varieties grown in Oregon, U.S.A.

S.No.	Variety	Type	% Amylose
1	Echo Russet	Russet	39.9
2	Gemstar	Russet	35.5
3	Russet Burbank	Russet	42.4
4	Classic Russet	Russet	44.1
5	Alturas	Russet	36.7
6	Clearwater Russet	Russet	36.8
7	Modoc	Red	41.8
8	Huckleberry Gold	Specialty	41.4
9	French Fingerling	Specialty	37.2
10	Waneta	Chip	41.8
11	Ruby Crescent	Specialty	35.2
12	Purple Fiesta	Specialty	35.8
13	Elfe	Specialty	38.7
14	Rainier Russet	Russet	30.3
15	Amarosa	Specialty	41.6
16	Ranger Russet	Russet	54.2
18	Red Rooster	Red	46.3
19	Shepody	Russet	47.0
20	Blushing Belle	Specialty	46.8
22	Russet Norkotah 296	Russet	32.3

## Data Availability

The data presented is contained within the article.
